# In-Plane Vibration Analysis of Annular Plates with Arbitrary Boundary Conditions

**DOI:** 10.1155/2014/653836

**Published:** 2014-01-28

**Authors:** Xianjie Shi, Dongyan Shi, Zhengrong Qin, Qingshan Wang

**Affiliations:** College of Mechanical and Electrical Engineering, Harbin Engineering University, Harbin 150001, China

## Abstract

In comparison with the out-of-plane vibrations of annular plates, far less attention has been paid to the in-plane vibrations which may also play a vital important role in affecting the sound radiation from and power flows in a built-up structure. In this investigation, a generalized Fourier series method is proposed for the in-plane vibration analysis of annular plates with arbitrary boundary conditions along each of its edges. Regardless of the boundary conditions, the in-plane displacement fields are invariantly expressed as a new form of trigonometric series expansions with a drastically improved convergence as compared with the conventional Fourier series. All the unknown expansion coefficients are treated as the generalized coordinates and determined using the Rayleigh-Ritz technique. Unlike most of the existing studies, the presented method can be readily and universally applied to a wide spectrum of in-plane vibration problems involving different boundary conditions, varying material, and geometric properties with no need of modifying the basic functions or adapting solution procedures. Several numerical examples are presented to demonstrate the effectiveness and reliability of the current solution for predicting the in-plane vibration characteristics of annular plates subjected to different boundary conditions.

## 1. Introduction

Annular plates are one of the most important structural components used in industrial applications and engineering fields. The dynamic characteristics of annular plates are thus of great interest to engineers and designers. Although there is a vast pool of studies about the out-of-plane vibrations of circular and annular plates [1], relatively few in-plane vibration results are reported for annular plates. In some practical engineering applications, however, the in-plane vibrations need to be taken into consideration. Furthermore, some studies have shown that the in-plane vibrations can play a prominent role in affecting the sound radiation and structural vibration in railway wheels, disk brakes, and hard disk drives [2, 3].

Love [4] first derived the essential equations of extensional motion and presented the general solution for a thin circular disk with free edge conditions. Then Onoe [5] obtained the in-plane displacements and frequency equations of circular disks based on Love's theory. In his study, he utilized trigonometric functions in the circumferential direction and Bessel functions in the radial direction. The dependence of nondimensional frequency parameters was examined on Poisson's ratio from 0.0 to 0.5. An error in the eigen-functions for compound modes was identified in his latter works [6]. Holland [7] used trigonometric and Bessel functions to study the in-plane vibration of a free circular plate. The frequency parameters for different Poisson's ratios were presented and compared with the results previously given by Onoe [5, 6]. Farag and Pan [8] examined the in-plane vibration of circular plates clamped at the outer edges. The mode functions were expressed in terms of trigonometric functions in the circumferential direction and the series expansion of Bessel functions in the radial direction. The frequency parameters were compared with finite element results and the data published previously. Recently, Park [9] derived exact frequency equations for in-plane vibration of a clamped circular plate using Hamilton's principle. His results were validated by finite element results and those from Farag and Pan [8]. Kim et al. [10] studied the in-plane vibration of a circular plate with its outer edge being restrained elastically. The mode shapes are expressed in terms of trigonometric functions in the circumferential direction and modal functions in the radial direction. The elastic boundary conditions at outer edge were represented by circumferentially distributed radial and tangential stiffness.

The above researches were limited to free in-plane vibrations of solid disks. Obtaining the in-plane vibration characteristics of circular annular plates has been treated in a few studies. Ambati et al. [11] proposed a generalized formulation for in-plane vibration analysis of annular disks and extended the method to solid disks as well as thin rings by varying size of the opening. The analytical results were verified through experiments. Irie et al. [12] used a transfer matrix method to study the in-plane vibration of circular and annular plates with free and clamped boundary conditions specified at the inner and outer edges, respectively. The frequency parameters were presented for circumferential wave numbers from 0 to 4. The circular plates were treated as a limiting case of an annular plate when the radius of the inner edge tends to zero. Using variational approach, Seok and Tiersten [13] investigated the in-plane vibration of a cantilevered annular sector plate where the plate is fixed on one radial edge and free on the rest. Singh and Muhammad [14] studied the free in-plane vibration of nonrectangular plate, including annular sector plates. Recently, Bashmal et al. [15] derived a generalized formula for in-plane vibration of circular annular plates under various combinations of classical boundary conditions. Ravari and Forouzen [16] derived the frequency equation for the in-plane vibration of orthotropic circular annular plates.

It should be pointed out that all the above studies are focused on annular and circular plate problems to which the solutions can be reduced to solving two coupled ordinary differential equations with respect to the radial dimension. When an analytical solution is sought for an annular plate, its boundary condition is typically limited to classical boundary conditions. An annular plate with arbitrary boundary condition is rarely attempted in the literature. Only Bashmal et al. [17] studied the in-plane vibration characteristics of annular plates with flexible boundary conditions at the inner and outer edges. In his study, the displacement fields were represented by trigonometric functions in the circumferential direction and by boundary characteristics orthogonal polynomials in the radial direction. However, annular plates with arbitrary boundary conditions have always been of research and application interests to many engineers [18–20]. In view of those technical limitations and practical needs, this investigation sets out to develop an analytical solution technique for the in-plane vibration analysis of annular plates with arbitrary boundary conditions. In principle, the method is similar to the improved Fourier series method (IFSM) originally proposed by Li [21] for the vibration of an arbitrarily supported beam, and subsequently extended to plates [22, 23], and cylindrical shells [24] and built-up structures [25, 26]. The displacement solutions, however, are here expressed as an accelerated trigonometric series expansion, rather than a Fourier series supplemented by polynomials. This modification is advantageous mathematically because of the “dual-invariance” of trigonometric functions under differential and integral operations. Numerical results are presented to verify the accuracy and reliability of the current solution method.

## 2. Theoretical Formulations

### 2.1. Descriptions of an Annular Plate

**Figure 1:**
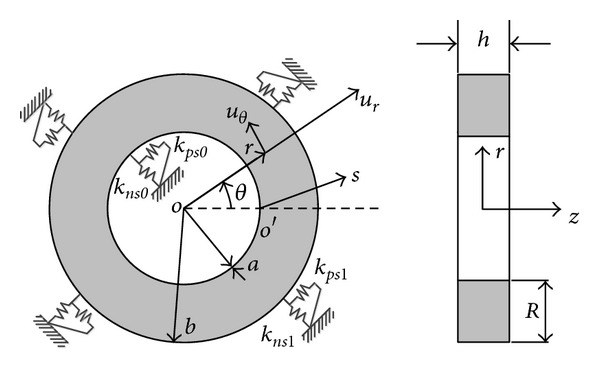
Sketch of an elastically restrained annular plate.

Figure 1 shows an annular plate with elastically restrained edges. This plate is of constant thickness *h*, inner radius *a*, outer radius *b*, and width *R* in radial direction. The plate geometry and dimensions are defined in a cylindrical coordinate system (*r*, *θ*, *z*). The displacement fields at a material point are denoted as *u*_*r*_ and *u*_*θ*_ in the radial and circumferential directions, respectively. The unit for displacement fields is m. A local cylindrical coordinate system, (*s*, *θ*, *z*), which is taken to describe the displacement fields and theoretical derivation, is also shown in Figure 1. The radial (*s*) and thickness (*z*) coordinates are measured normally from the inner edge and the midplane of the annular plate, respectively, and *θ* is the circumferential angle. The annular plate can be accordingly defined as

(1) 0≤s≤R(≡b−a),−h2≤z≤h2,0≤θ≤2π.

The boundary conditions for in-plane motion can be generally specified in terms of two types of restraining springs (normal and tangential) with independent stiffness values along each edge. Classical boundary conditions and their combinations can be readily realized by setting the spring stiffness of restraint springs to zero or an extremely large number. Also the general elastic boundary supports can be physically achieved with different spring constants. For instance, a free edge condition is simply created by setting the stiffness values for the two sets of springs to zero. The unit for the stiffness of the distributed (tangential or normal) spring is N/m^2^, rather than N/m.

### 2.2. Series Representations of the Displacement Functions

Regardless of the boundary conditions, the displacement functions will be invariably sought as trigonometric series expansions in the form of

(2) $$\begin{array}{c} u_{r}\left(s,\theta \right)=\overset{\infty }{\underset{m=-2}{\sum }}\;\overset{\infty }{\underset{n=0}{\sum }}\varphi_{m}\left(s\right)\left[A_{mn}\cos\left(n\theta \right)+B_{mn}\sin\left(n\theta \right)\right],\\ u_{\theta }\left(s,\theta \right)=\overset{\infty }{\underset{m=-2}{\sum }}\;\overset{\infty }{\underset{n=0}{\sum }}\varphi_{m}\left(s\right)\left[C_{mn}\cos\left(n\theta \right)+D_{mn}\sin\left(n\theta \right)\right], \end{array}$$

where *A*_*mn*_, *B*_*mn*_, *C*_*mn*_, and *D*_*mn*_ denote the Fourier coefficients of series expansions, and

(3) φm(s)={cos⁡λms,m≥0,sinλms,m<0, λm=mπR.

It should be pointed out that the displacements in (2) are expanded according to symmetric and antisymmetric circumferential modes. The above series expansions without sine terms in radial direction will constitute a complete set which is adequate to span a vector space of infinite dimensions. Thus, the displacement solution can be considered as a special vector (in this space) which satisfies both the governing differential equations and the boundary conditions for the plate problem. The number of sine terms in radial direction is dictated by the desired degree of smoothness with the solution. Since the in-plane displacements are governed by second-order differential equations over the plate, their first derivatives are mathematically required to be continuous and second derivatives exist everywhere. By including two sine terms in radial direction (corresponding to *m* = −2 and −1 in (2)), these series are able to expand and uniformly converge to *any* function*f*(*s*, *θ*) ∈ **C** ^1^ for all (*s*, *θ*) ∈ *D* : ([0, *R*]×[0, 2*π*]). The negatively indexed expansion coefficients are determined only by the first derivatives at the boundaries of the solution domain. Strictly speaking, the sine terms in radial direction are “redundant” in view of the fact that the basis set (i.e., the series expansions without sine terms in radial direction) is complete. More explicitly, the negatively indexed coefficients can be shown to be dependent on those for the cosine series under the condition that the (cosine) series converges fast enough so that it can be differentiated term by term. In such a case, for example, the relationships between the coefficients with negative and nonnegative indexes can be established through the boundary conditions. A strong form of solution can be subsequently obtained by letting the series satisfy the governing differential equations on a point-wise basis (i.e., at each field point). Such a solution may be considered exact in the sense that the solution error can be controlled within any prespecified tolerance.

In seeking an approximate solution, the truncated version of the series expansions will have to be adopted. As a result, all the expansion coefficients, including those with negative index, will be treated equally as the generalized coordinates and solved from, for example, the Rayleigh-Ritz procedure. It shall be noted that since the trial solution is constructed with the same degree of smoothness as required for a strong solution, the approximate and exact solutions are equivalent mathematically (this is evident from integrating, by part, the appropriate energy equation). In terms of numerical implementations, however, the approximate solution is far more advantageous, especially when the modeling method is extended to structures involving many plates or other structural elements.

### 2.3. Solution for the Annular Plate

For small deformations, the strain-displacement relations in local cylindrical coordinate system (*s*, *θ*, *z*) are given by [9]

(4) $$\begin{array}{c} \varepsilon_{s}=\frac{\partial u_{r}}{\partial s},\\ \varepsilon_{\theta }=\frac{1}{s+a}\left(u_{r}+\frac{\partial u_{\theta }}{\partial \theta }\right),\\ \varepsilon_{s\theta }=\frac{1}{s+a}\frac{\partial u_{r}}{\partial \theta }+\frac{\partial u_{\theta }}{\partial s}-\frac{u_{\theta }}{s+a}. \end{array}$$

The strain energy *V*_*p*_ of the deformed plate is given as [9]

(5) Vp=  12∫0R∫02π∫−h(s,θ)/2h(s,θ)/2(σsεs+σθεθ+σsθεsθ)         ×(s+a)dz dθ ds.

The normal and shear stresses in (5) can be calculated from [15]

(6) $$\begin{array}{c} \sigma_{s}=\frac{E\left(s,\theta \right)}{1-v^{2}\left(s,\theta \right)}\left(\varepsilon_{s}+v\left(s,\theta \right)\varepsilon_{\theta }\right),\\ \sigma_{\theta }=\frac{E\left(s,\theta \right)}{1-v^{2}\left(s,\theta \right)}\left(v\left(s,\theta \right)\varepsilon_{s}+\varepsilon_{\theta }\right),\\ \sigma_{s\theta }=\frac{E\left(s,\theta \right)}{2\left(1+v\left(s,\theta \right)\right)}\varepsilon_{s\theta }, \end{array}$$

where *E*(*s*, *θ*) and *v*(*s*, *θ*) are, respectively, Young's modulus and Poisson's ratio which can vary with spatial coordinates.

Submitting (4) and (6) into (5) will lead to the following expression for the strain energy:

(7) Vp=12∫0R∫02πG(s,θ)[(∂ur∂s)2+(urs+a)2+2v(s,θ)×(urs+a∂ur∂s+1s+a∂uθ∂θ∂ur∂s)+2ur(s+a)2∂uθ∂θ+1(s+a)2(∂uθ∂θ)2+1−v(s,θ)2×(1s+a∂ur∂θ+∂uθ∂s−uθs+a)2]     ×(s+a)dθ ds,

where *G*(*s*, *θ*) = *E*(*s*, *θ*)*h*(*s*, *θ*)/(1 − *v* ^2^(*s*, *θ*)) is the extensional rigidity of the plate.

By neglecting rotary inertia, the kinetic energy of an annular plate can be written as

(8) $$\begin{array}{c} T=\frac{1}{2}\omega^{2}\overset{R}{\underset{0}{\int }}\overset{2\pi }{\underset{0}{\int }}\rho \left(s,\theta \right)h\left(s,\theta \right)\left(u_{r}^{2}+u_{\theta }^{2}\right)\left(s+a\right)d\theta \;ds, \end{array}$$

where *ω* is the frequency in radians; *ρ*(*s*, *θ*) is the material density which can vary with spatial coordinates.

In seeking a weak form of solution, the presence of the restraining springs can be easily accounted for in terms of potential energies stored in the boundary springs

(9) Vbc=12∫02π(kns0(θ)ur2+kps0(θ)uθ2︸s=0)  a dθ +12∫02π(kns1(θ)ur2+kps1(θ)uθ2︸s=R)b dθ.

The definitions of stiffness functions are given in the Nomenclature.

The Lagrangian function for an annular plate can be finally expressed as

(10) $$\begin{array}{c} L=V_{p}+V_{bc}-T. \end{array}$$

In the above derivations, the thickness of the plate, material properties (Young's modulus, Poisson's ratio, and mass density) and the stiffness for each elastic restraint are all generally specified as an arbitrary function of the spatial coordinates. To unify the descriptions and facilitate the analytical calculations of the involved integrals, all these distribution functions can be expanded into 1-D or 2-D Fourier cosine series.

By substituting the displacement functions (2) into the Lagrangian (10) and minimizing the result with respect to all the unknown Fourier coefficients, one is able to yield a final system of equation as

(11) $$\begin{array}{c} \left(K-\omega^{2}M\right)E=0, \end{array}$$

where **K** and **M** are the stiffness and mass matrices defined as

(12) $$\begin{array}{c} K=\left[\begin{array}{cccc} K_{1-1} & K_{1-2} & K_{1-3} & K_{1-4}\\ K_{2-1} & K_{2-2} & K_{2-3} & K_{2-4}\\ K_{3-1} & K_{3-2} & K_{3-3} & K_{3-4}\\ K_{4-1} & K_{4-2} & K_{4-3} & K_{4-4} \end{array}\right],\\ M=\left[\begin{array}{cccc} M_{1-1} & M_{1-2} & M_{1-3} & M_{1-4}\\ M_{2-1} & M_{2-2} & M_{2-3} & M_{2-4}\\ M_{3-1} & M_{3-2} & M_{3-3} & M_{3-4}\\ M_{4-1} & M_{4-2} & M_{4-3} & M_{4-4} \end{array}\right] \end{array}$$

and **E** is a vector of all the unknown Fourier expansion coefficients; that is,

(13) E={A−2,0,A−2,1,…,A−2,N,…,Am,0,…,  Am,n,…,AM,N,B−2,1,B−2,2,…,  B−2,N,…,Bm,1,…,Bm,n,…,BM,N,  C−2,0,C−2,1,…,C−2,N,…,  Cm,0,…,Cm,n,…,CM,N,  D−2,1,D−2,2,…,D−2,N,…,  Dm,1,…,Dm,n,…,DM,N}T.

The natural frequencies and eigenvectors of an annular plate can now be easily and directly determined from solving a standard matrix eigenvalue problem, (11). For a given natural frequency, the corresponding eigenvector actually contains the Fourier coefficients which can be used to construct the physical mode shape based on (2). Although this investigation is focused on the free in-plane vibration of annular plates, the dynamic response of the plates to any applied load can be easily considered by including the work done by this load in the Lagrangian, which will eventually lead to a force term on the right side of (11).

It should also be noted that the current method is particularly advantageous in obtaining other variables of interest such as power flows. Since the displacements are constructed sufficiently smooth as required in a strong formulation, postprocessing the solution can be done easily through appropriate mathematical operations, including term-by-term differentiations.

## 3. Results and Discussions

Several numerical examples will be presented in this section to demonstrate the accuracy and reliability of the proposed method. Throughout these examples, the mass density, Young's modulus, and Poisson's ratio are specified as *ρ* = 7800 kg/m^3^, *E* = 2.1 × 10^11^ Pa, and *v* = 0.3. In identifying the boundary conditions, letters C and F have been used to indicate the clamped and free boundary conditions along an edge, respectively. Thus, the boundary conditions for a plate are fully specified by using two letters with the first one indicating the B.C. along the inner radius edge, *r* = *a*. The second one is specified for the edge, *r* = *b*.

First, consider an annular plate which is fully clamped (C-C) along each edge. A clamped edge refers to a special case of the elastic supports when the stiffness for both of the (normal and tangential) restraining springs becomes infinity (represented by a very large number, 10^13^, in the actual calculations).  Table 1 presents the first dimensionless frequency parameters, *Ω* = *ωb*(*ρ*(1 − *v* ^2^)/*E*)^1/2^, for C-C annular plates with various cutout ratios and circumferential wave numbers. The frequency parameter with *n* = 0 is the torsional vibration frequency parameter. In the subsequent tables, the *n* = 0 also represents the torsional vibration frequency parameter. The reference results obtained from [12, 15, 16] are also given there for comparisons. The results obtained by the present method are in good agreement with those of other authors, which indicates the accuracy of this method. As mentioned earlier, the series expansions, (2), will have to be truncated in numerical calculations. Specifically, the frequency parameters in Table 1 are determined by truncating the series at *M* = *N* = 14. To check the convergence of the solution, Table 2 shows the frequency parameters calculated by using different numbers of expansion terms, *M* = *N* = 6, 7, …, 15. A good convergence behavior is observed. Of equal importance, the solution also shows an excellent numerical stability, meaning that the values essentially remain the same as the truncation numbers become increasingly large. Once the convergence behavior is understood, the series will be consistently truncated to *M* = *N* = 14 in all the subsequent calculation.

Next example concerns completely free annular plates (F-F), which represents a classical, but quite challenging, case for validating the solution. Under the current framework, the free edge condition is easily realized by setting all the stiffness constants to zero. The first frequency parameters, *Ω* = *ωb*(*ρ*(1 − *v* ^2^)/*E*)^1/2^, are presented in Table 3 for each of the wave numbers *n* and different cutout ratios. Three sets of reference results are also given there for comparisons. These four sets of solutions agree well with each other.

**Figure 2:**
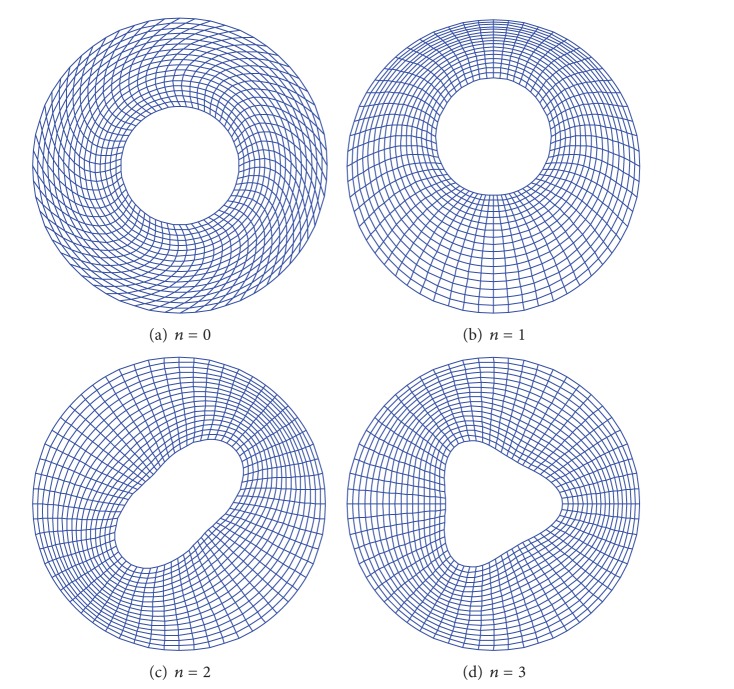
Mode shapes for F-C annular plate (*a*/*b* = 0.4) with *n* = 0, 1, 2, and 3.

To further validate the accuracy and reliability of the presented method, two more classical cases (C-F and F-C) are also considered, and the corresponding frequency parameters are presented in Tables 4 and 5, respectively. In both cases, the current results agree well with those taken from [12, 15, 16]. For any given modal frequency, the corresponding mode shape can be readily determined from (2). The first four mode shapes (*n* = 0, 1, 2, and 3) are plotted in Figure 2 for the F-C annular plate with cutout ratio *a*/*b* = 0.4. It is seen that although those are the lower-order modes, they tend to exhibit unfamiliar and more complicated patterns than their counterparts in the flexural vibrations. For instance, the extension-compression deformation in one mode (or region) can quickly turn into a shear state in another mode (or region). This characteristic, however, may have some useful implications to the nondestructive evaluation of material and structural parameters or monitoring of structural conditions or failures, as evidenced by the more distinctively different modal signatures and more probing natures of the in-plane displacement fields. The complexity of mode shapes also graphically confirms the fact that the displacement fields can no longer be determined by the separation of variables for a plate under general boundary condition.

In the literature, there are two distinct types of “simply support” boundary conditions for the in-plane vibration of a plate [27]. For convenience, these two types of simply supported conditions are designated by SS1 and SS2. An edge associated with the first type, SS1, is characterized by the fact that the plate displacement parallel to the edge is specified to zero as well as normal stress perpendicular to the edge. In the numerical calculation, it can be physically realized by setting the stiffness of the tangential and normal springs to *∞* and 0, respectively. The second type is an exactly opposite scenario.

The first frequency parameters, *Ω* = *ωb*(*ρ*(1 − *v* ^2^)/*E*)^1/2^, are given in Table 6 for C-SS1 annular plates with clamped and simply supported (SS1) along inner and outer edge, respectively. To understand the difference between the SS1 and SS2 boundary conditions, the problems are resolved by only replacing the SS1 conditions with SS2 while all the other parameters are kept the same. The results are shown in Table 7. A comparison of results in Tables 6 and 7 has revealed the noticeable difference between the SS1 and SS2 boundary conditions, as manifested in values of the frequency parameters. The frequency parameters monotonically increase for these two boundary conditions.

Traditionally, the displacement expressions and the subsequent solution algorithms and implementations are dictated by the intended boundary condition. Consequently, most studies are specifically related to a particular type of boundary conditions. In the above examples, it has been demonstrated that the proposed analytical method can be universally applied to different boundary conditions with no need of making any algorithm or procedural modifications; the modifying boundary conditions is as simple as changing plate parameters such as geometrical and material properties. Consider the simply supported case (SS1-SS1) for example. It can be produced easily by letting the stiffness of the tangential and normal springs to *∞* and 0, respectively.  Table 8 shows the first frequency parameters for SS1-SS1 annular plate with different cutout ratios.

**Figure 3:**
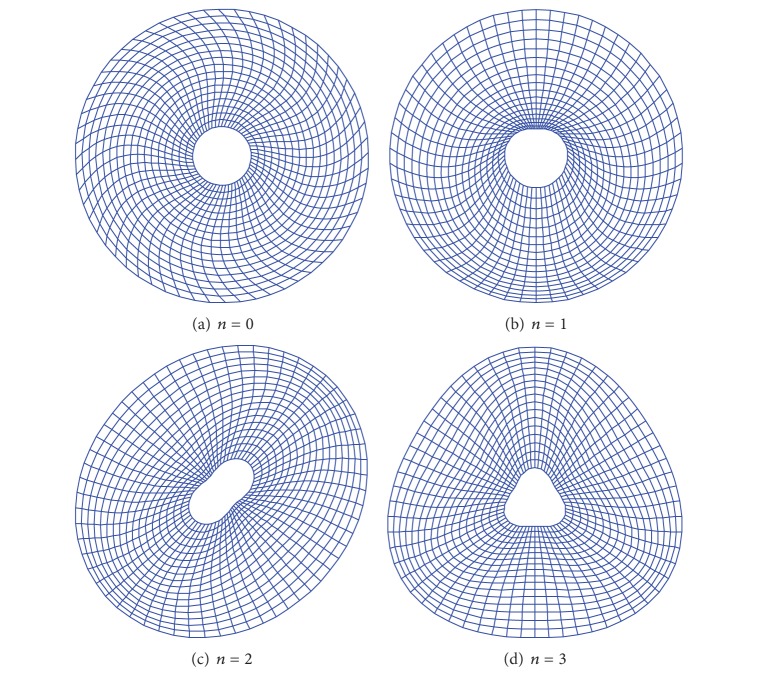
Mode shapes for elastic restrained annular plate (*a*/*b* = 0.2) with *n* = 0, 1, 2, and 3.

All the examples considered thus far have been limited to the classical boundary conditions which are viewed as the special cases of elastically restrained edges. We now turn to elastically restrained plates. The first one involves a simply supported (SS1-SS1) annular plate (*a*/*b* = 0.4) with a uniform normal restraint along each edge. The calculated frequency parameters are given in Table 9 together with the FEM results. The second example concerns a plate elastically supported along all edges. The stiffness values for the normal and tangential restraints are both set equal to 10^9^ N/m^2^. Several frequency parameters are shown in Table 10. Since elastically restrained plates are rarely investigated, the FEM results are used as the reference. A good agreement is observed between the current and the FEM solutions. The mode shapes for annular plate with cutout ratio *a*/*b* = 0.2 are plotted in Figure 3. Again, the plots in Figure 3 illustrate the complexities of in-plane modes or vibrations.

**Figure 4:**
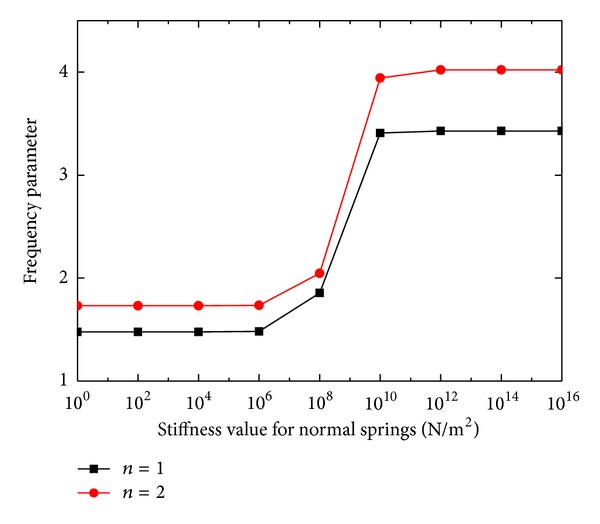
The effects of normal boundary spring stiffness on frequency parameters.

**Figure 5:**
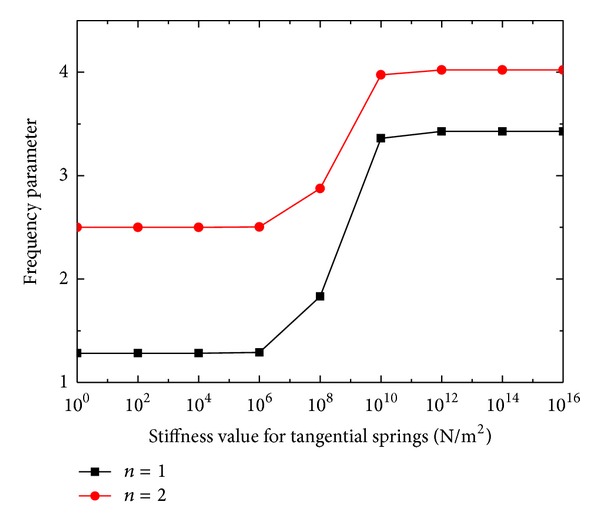
The effects of tangential boundary spring stiffness on frequency parameters.

Finally, the effects of stiffness of boundary springs on frequency parameters for annular plate (*a*/*b* = 0.4) are investigated, with corresponding results shown in Figures 4 and 5. The curves indicating the frequency parameters for *n* = 1 and 2 are obtained by varying the stiffness of one group of the boundary springs from 10^16^ to 10^0^ while assigning the other groups of springs with 10^16^.

The curves in Figures 4 and 5 can clearly tell how the frequency parameters for *n* = 1 and 2 can be modified by the restraining springs for a large stiffness range from 1 to 10^16^ N/m^2^. It is noticed that the frequency parameters almost keep at a level when the stiffness of the restraining springs is larger than 10^10^ or smaller than 10^6^. Within the stiffness range [10^6^, 10^10^], the frequency parameters change greatly. Essentially, the edge can be considered free (fixed) if the stiffness for the restraining spring is smaller (larger) than 10^6^ (10^10^). Thus, it is suitable to use 10^13^ to simulate the infinite stiffness value in the validation part.

## 4. Conclusions

A general analytical method is presented for the free in-plane vibration analysis of annular plates with general elastic boundary supports along each edge. The in-plane displacement fields are simply and invariably expressed as a new form of trigonometric series expansions which converge uniformly and polynomially over the solution domain. These Fourier sine functions are introduced to deal with the potential discontinuities (or jumps) at the edges with the partial derivatives of the in-plane displacements. Unlike most of the existing methods in which each of the frequency parameters is calculated, repeatedly and iteratively, from a nonlinear characteristic equation, all the modal parameters can now be easily and simultaneously determined from solving a standard matrix eigenvalue problem. The current method provides a unified solution to a wide range of plate problems involving different boundary conditions and complicating factors. In comparison with most existing techniques, the current one does not require any formulation or implementation modifications to accommodate different boundary conditions; all the familiar homogeneous boundary conditions are simply considered as the special cases when the stiffness values of each restraining springs are zero or infinity.

The excellent accuracy and convergence of the present solution method have been checked through numerical comparisons with finite element solution and/or results existing in literatures. Good agreements observed from all the cases verify that the current method can serve as a reliable tool for the in-plane vibration analysis of annular plates with general boundary supports and also provides benchmark solution for the developments of other modeling methods or relevant vibration engineering software in the future. It also should be pointed out that this solution method is readily applicable to plates with nonuniform stiffness, thickness distributions, and varying material properties.

## Figures and Tables

**Table 1:** Frequency parameters, Ω = *ωb*(*ρ*(1 − *v* ^2^)/*E*)^1/2^, for C-C annular plates with various cutout ratios.

*a*/*b*	Circumferential wave number *n*				
	0	1	2	3	4
0.2	2.5059	2.7833	3.3778	4.0655	4.8010
	(2.506)^a^	(2.783)	(3.378)	(4.066)	(4.802)
	—	(2.806)^b^	(3.394)	(4.084)	(4.811)
	—	(2.886)^c^	(3.483)	(4.119)	(4.916)
0.4	3.1895	3.4292	4.0224	4.7068	5.2871
	(3.189)^a^	(3.429)	(4.023)	(4.707)	(5.287)
	—	(3.456)^b^	(4.046)	(4.737)	(5.360)
	—	(3.532)^c^	(4.130)	(4.766)	(5.470)
0.6	4.6915	4.8345	5.2365	5.8319	6.5414
	(4.692)^a^	(4.835)	(5.237)	(5.832)	(6.541)
	—	(4.939)^c^	(5.339)	(5.893)	(6.650)
0.8	9.3104	9.3702	9.5473	9.8349	10.223
	(9.310)^a^	(9.370)	(9.547)	(9.835)	(10.223)
	—	(9.476)^c^	(9.658)	(9.898)	(10.432)

^a^Results in parentheses are taken from [12].

^b^Results in parentheses are taken from [15].

^c^Results in parentheses are taken from [16].

**Table 2:** Convergence study for frequency parameters Ω = *ωb*(*ρ*(1 − *v* ^2^)/*E*)^1/2^ for a C-C annular plate (*a*/*b* = 0.4).

	Circumferential wave number *n*				
	0	1	2	3	4
*M* = *N* = 6	3.1895	3.4292	4.0227	4.7586	5.3064
*M* = *N* = 7	3.1895	3.4292	4.0225	4.7093	5.3064
*M* = *N* = 8	3.1895	3.4292	4.0225	4.7086	5.3064
*M* = *N* = 9	3.1895	3.4292	4.0224	4.7071	5.2954
*M* = *N* = 10	3.1895	3.4292	4.0224	4.7070	5.2945
*M* = *N* = 11	3.1895	3.4292	4.0224	4.7069	5.2893
*M* = *N* = 12	3.1895	3.4292	4.0224	4.7069	5.2884
*M* = *N* = 13	3.1895	3.4292	4.0224	4.7068	5.2871
*M* = *N* = 14	3.1895	3.4292	4.0224	4.7068	5.2871
*M* = *N* = 15	3.1895	3.4292	4.0224	4.7068	5.2870
[12]	3.189	3.429	4.023	4.707	5.287
FEM	3.1912	3.4311	4.0249	4.7100	5.2917

**Table 3:** Frequency parameters, Ω = *ωb*(*ρ*(1 − *v* ^2^)/*E*)^1/2^, for F-F annular plates with various cutout ratios.

*a*/*b*	Circumferential wave number *n*				
	0	1	2	3	4
0.2	3.0892	1.6512	1.1100	2.0710	2.7671
	(3.090)^a^	(1.652)	(1.110)	(2.071)	(2.767)
	—	(1.651)^b^	(1.111)	(2.072)	(2.766)
	—	(1.661)^c^	(1.120)	(2.074)	(2.768)
0.4	3.5295	1.6824	0.7214	1.6188	2.4832
	(3.530)^a^	(1.683)	(0.721)	(1.618)	(2.482)
	—	(1.682)^b^	(0.721)	(1.619)	(2.482)
	—	(1.700)^c^	(0.727)	(1.623)	(2.485)
0.6	4.8673	1.6178	0.4182	1.0432	1.7528
	(4.867)^a^	(1.618)	(0.418)	(1.043)	(1.752)
0.8	9.3802	1.4879	0.1784	0.4873	0.8935
	(9.380)^a^	(1.488)	(0.178)	(0.487)	(0.892)

^a^Results in parentheses are taken from [12].

^b^Results in parentheses are taken from [15].

^c^Results in parentheses are taken from [16].

**Table 4:** Frequency parameters, Ω = *ωb*(*ρ*(1 − *v* ^2^)/*E*)^1/2^, for C-F annular plates with various cutout ratios.

*a*/*b*	Circumferential wave number *n*				
	0	1	2	3	4
0.2	0.3524	0.9194	1.5415	2.1569	2.7782
	(0.352)^a^	(0.919)	(1.542)	(2.157)	(2.778)
	—	(0.940)^b^	(1.561)	(2.166)	(2.779)
	—	(0.928)^c^	(1.548)	(2.163)	(2.781)
0.4	0.8235	1.2811	1.9644	2.4451	2.9112
	(0.823)^a^	(1.281)	(1.965)	(2.445)	(2.911)
	—	(1.296)^b^	(1.982)	(2.463)	(2.924)
	—	(1.297)^c^	(1.969)	(2.469)	(2.926)
0.6	1.6623	1.9519	2.6077	3.296	3.7228
	(1.662)^a^	(1.952)	(2.608)	(3.294)	(3.722)
0.8	4.0377	4.1611	4.5102	5.0359	5.6841
	(4.038)^a^	(4.161)	(4.510)	(5.036)	(5.684)

^a^Results in parentheses are taken from [12].

^b^Results in parentheses are taken from [15].

^c^Results in parentheses are taken from [16].

**Table 5:** Frequency parameters, Ω = *ωb*(*ρ*(1 − *v* ^2^)/*E*)^1/2^, for F-C annular plates with various cutout ratios.

*a*/*b*	Circumferential wave number *n*				
	0	1	2	3	4
0.2	2.2840	2.1033	2.5532	3.6888	4.7121
	(2.284)^a^	(2.104)	(2.553)	(3.688)	(4.712)
	—	(2.106)^b^	(2.556)	(3.693)	(4.718)
	—	(2.107)^c^	(2.561)	(3.700)	(4.721)
0.4	2.4639	2.5169	2.7206	3.2142	3.9565
	(2.464)^a^	(2.517)	(2.721)	(3.214)	(3.955)
	—	(2.522)^b^	(2.734)	(3.219)	(3.960)
	—	(2.601)^c^	(2.739)	(3.219)	(3.961)
0.6	3.0836	3.2604	3.6299	3.9422	4.2507
	(3.084)^a^	(3.260)	(3.630)	(3.942)	(4.250)
0.8	5.2948	5.4017	5.7077	6.1738	6.7411
	(5.295)^a^	(5.402)	(5.708)	(6.174)	(6.741)

^a^Results in parentheses are taken from [12].

^b^Results in parentheses are taken from [15].

^c^Results in parentheses are taken from [16].

**Table 6:** Frequency parameters, Ω = *ωb*(*ρ*(1 − *v* ^2^)/*E*)^1/2^, for C-SS1 annular plates with various cutout ratios.

*a*/*b*	Circumferential wave number *n*				
	0	1	2	3	4
0.2	2.5059	1.5923	1.6172	2.1571	2.8112
	(2.5066)^a^	(1.5923)	(1.6172)	(2.1575)	(2.8121)
0.4	3.1895	2.3526	2.2122	2.4531	2.9322
0.6	4.6915	3.7429	3.5579	3.5515	3.7324
0.8	9.3104	7.7222	7.6007	7.4883	7.4254

^a^Results in parentheses are calculated using ABAQUS.

**Table 7:** Frequency parameters, Ω = *ωb*(*ρ*(1 − *v* ^2^)/*E*)^1/2^, for C-SS2 annular plates with various cutout ratios.

*a*/*b*	Circumferential wave number *n*				
	0	1	2	3	4
0.2	4.2357	1.3344	2.4986	3.4605	4.2533
	(4.2369)^a^	(1.3344)	(2.4993)	(3.4629)	(4.2578)
0.4	5.3911	1.4648	2.5258	3.5715	4.4481
0.6	6.8087	2.0057	2.7866	3.7234	4.6936
0.8	13.760	4.1684	4.5373	5.0913	5.7751

^a^Results in parentheses are calculated using ABAQUS.

**Table 8:** Frequency parameters, Ω = *ωb*(*ρ*(1 − *v* ^2^)/*E*)^1/2^, for SS1-SS1 annular plates with different cutout ratios.

*a*/*b*	Circumferential wave number *n*				
	0	1	2	3	4
0.2	2.5059	1.3570	1.5314	2.1413	2.8090
	(2.5066)^a^	(1.3560)	(1.5306)	(2.1413)	(2.8097)
0.4	3.1895	1.4782	1.7312	2.2297	2.8384
0.6	4.6915	1.3825	1.7869	2.3140	2.8999
0.8	9.3104	1.2448	1.6725	2.2081	2.7904

^a^Results in parentheses are calculated using ABAQUS.

**Table 9:** Frequency parameters, Ω = *ωb*(*ρ*(1 − *v* ^2^)/*E*)^1/2^, for SS1-SS1 annular plates (*a*/*b* = 0.4) with different cutout ratios.

*K* (N/m^2^)	Circumferential wave number *n*				
	0	1	2	3	4
10^0^	3.1895	1.4782	1.7312	2.2297	2.8384
	(3.1962)^a^	(1.4788)	(1.7324)	(2.2319)	(2.8424)
10^4^	3.1895	1.4782	1.7312	2.2298	2.8384
10^8^	3.1895	1.8551	2.0465	2.4938	3.0716
10^12^	3.1895	3.4290	4.0217	4.7051	5.2852

^a^Results in parentheses are calculated using ABAQUS.

**Table 10:** Frequency parameters, Ω = *ωb*(*ρ*(1 − *v* ^2^)/*E*)^1/2^, for an annular plate with identical restraints at all edges: *K* = 10^9^ N/m^2^.

*a*/*b*	Circumferential wave number *n*				
	0	1	2	3	4
0.2	2.1902	2.1652	2.5510	3.2580	3.9685
	(2.1913)^a^	(2.1663)	(2.5518)	(3.2610)	(3.9743)
0.4	2.5972	2.6686	2.9584	3.4023	3.9728
	(2.6000)^a^	(2.6912)	(2.9605)	(3.4065)	(3.9797)
0.6	3.4482	3.5532	3.7939	4.0784	4.4150
0.8	5.5242	5.5974	5.7866	6.0011	6.1803

^a^Results in parentheses are calculated using ABAQUS.

## Nomenclature

*a*, *b*: Inner radius and outer radius of plate
*R*: Width of plate in radial direction
*h*: Thickness of plate
*E*: Young's modulus
*ρ*: Mass density
*G*: Extensional rigidity
*v*: Poisson's ratio
*u*_*r*_, *u*_*θ*_: Radial and circumferential displacements, respectively, along *r* and *θ* directions
*k*_*ps*0_, *k*_*ps*1_: Stiffness for tangential springs, respectively, at *s* = 0 and *R*
*k*_*ns*0_, *k*_*ns*1_: Stiffness for normal springs, respectively, at *s* = 0 and *R*
*A*_*mn*_, *B*_*mn*_, *C*_*mn*_, *D*_*mn*_: Fourier series coefficients
*V*_*p*_: Strain energies due to in-plane motion
*V*_*bc*_: Potential energies stored in boundary springs of the plate
*T*: Total kinetic energy
*L*: Lagrangian operator
*ω*: Frequency in radian
**K**: Stiffness matrix
**M**: Mass matrix
**E**: Vector of Fourier coefficients
*M*, *N*: Truncation number
*Ω*: Dimensionless frequency parameter.
